# Air Travel and the Spread of Influenza: Authors' Reply

**DOI:** 10.1371/journal.pmed.0030502

**Published:** 2006-11-28

**Authors:** John S Brownstein, Kenneth D Mandl, Cecily J Wolfe

**Affiliations:** Children's Hospital Boston, Harvard–MIT Division of Health Sciences and Technology Harvard Medical School Boston, Massachusetts, United States of America; University of Hawaii at Manoa, Honolulu, Hawaii, United States of America

Viboud et al. [1] offer thoughtful commentary on our paper [2], opening a scientific exchange that we hope brings attention to a critical issue. We reported the first empirical and quantitative evidence for the effect of airline travel on the rate of epidemic influenza spread. Though other investigators have also found this relationship [3–6], there is no consensus on effect size. We welcome scrutiny of our methods and results and believe the findings stand for the following reasons.

Viboud et al., taking a longer historical perspective, suggest that the slower spread and late peaking of the 2001–2002 season is not unique and highlight three other late seasons dominated by influenza B (1992–1993–1973–1974, and 1976–1977). Recent studies, including one by Viboud and colleagues, find that B seasons have different epidemiological characteristics than A/H3N2 seasons, which may explain the late peaking in these years [7,8]. As 2001–2002 was dominated by influenza A/H3N2, late peaking in 2001–2002 cannot be explained by dominant subtype (nor climatic conditions). Our study period from 1996–2005 represents the longest stretch of A/H3N2 seasons in over 30 years, essentially controlling for subtype. Viboud et al. also point to the 2005–2006 season as particularly delayed. We examined peaking during that season using mortality data from the US Centers for Disease Control and Prevention, in order to compare to our estimates from prior seasons [9]. We find that mortality as well as morbidity was bimodal, with larger peaks in January and December, respectively. We thus reaffirm that 2001–2002, being the latest peaking H3N2 season in over 30 years, is an aberrant season for which airline travel interruption remains the best explanation.

**Figure 1:**
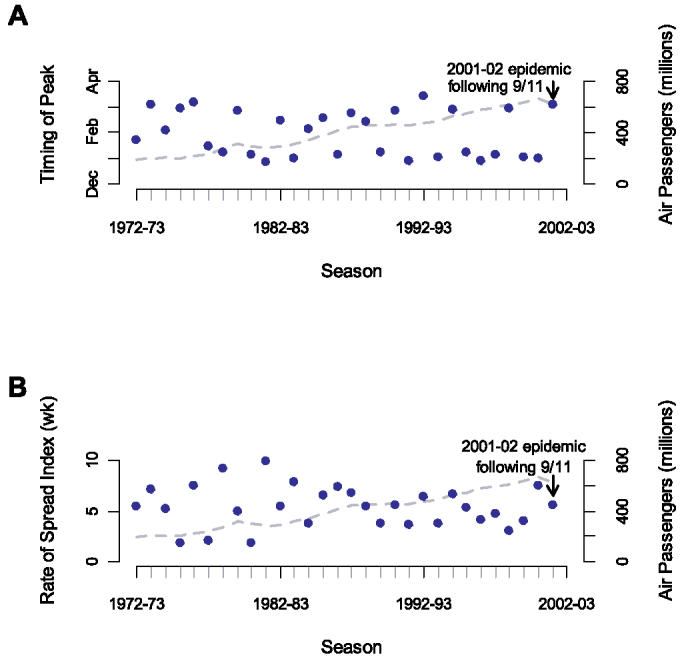
Influence of United States Airline Volume on Influenza Spread and the Timing of Yearly Transmission over 30 Influenza Seasons (A) The association between airline travel from September to December and the timing of the US influenza peak is displayed. Airline volume significantly predicts the timing of seasonal influenza mortality (*r* *^2^* = 0.460; *p* < 0.001). The timing of seasonal national influenza mortality (blue dots) is estimated from cross-correlation of each season with the 1972–1973 season. (B) The association between airline travel from September to December and time to transnational US spread is displayed. The numbers of traveling domestic passengers significantly predicts transnational influenza spread (*r* *^2^* = 0.265; *p* = 0.004). Index spread of influenza (blue dots) is estimated from the 99% confidence interval of the cross-correlation values for the nine major geographic regions of the United States against the national curve for a give year, as previously described [2].

Viboud and colleagues' letter does not take into account that our results are not simply based on an outlier year, nor are they based on a single data source. Rather, we have revealed an important correlation across nine influenza seasons. The impact of airline volume on flu spread does not depend on the 2001–2002 season and remains significant even after its exclusion. Since considering longer time series may provide insight, we repeated our methods on the 30-year mortality time series which Viboud et al. also analyzed [10]. We employed the same spatial aggregation (nine geographic regions) and time series methods as described in [2]. We found substantial long-term log-linear trends toward earlier peaking and faster spreading influenza epidemics that are correlated with air travel volume (*r* *^2^* = 0.460; *p* < 0.001 and *r* *^2^* = 0.265; *p* = 0.004, respectively, Figure 1). Thus, our new analyses of longer-term data support an effect of airline travel volume.

We strongly caution that other factors may influence these trends, including population density, air passenger demographics, ground transportation, and climate. Our design relies on a shorter, more recent time series to avoid confounding by longer term secular trends that may be evident in the 30-year time series. Given the three year backlog of the 30-year dataset, the data Viboud et al. use do not permit the interrupted time series analysis at the core of our investigation.

Reconciliation of our different time series methodologies and datasets should be considered in future research. Nonetheless, because our results were confirmed with viral surveillance data, we remain confident in the robustness of our analysis. We agree that other modes of transportation are important influences; our paper makes no claim that air travel is the only mechanism of spread, and we explicitly report that our model explains a portion of the variation in yearly influenza spread and peak.

Finally, Viboud et al. emphasize the limited applicability of our findings to pandemics. We agree and have highlighted this limitation in our paper. The decision to restrict travel should be multifactorial. We do hope that it will be evidence based. Our analyses (including those presented here) provide empirical insight into the previously uncharacterized effect of air travel fluctuation on influenza spread. They are one contribution to a small body of investigations that are forming the basis of global policy on flu preparedness. Though the effect we observe might be smaller under pandemic conditions, the benefit of a delay is worthy of consideration by scientists and policy makers where lives are at stake and even a short lead time may be of enormous public health value.

We are pleased that Viboud et al. have engaged in a discourse that we hope will strengthen the scientific basis of pandemic preparedness. We call on governments, industry, and health care to create a more accessible, freely available, and well-documented data repository for geographically and temporally detailed data on influenza [11] and encourage empirical analyses of the dynamics and mechanisms of influenza spread.
