# Correction: Air Travel and the Spread of Influenza: Authors' Reply

**DOI:** 10.1371/journal.pmed.0040033

**Published:** 2007-01-30

**Authors:** John S Brownstein, Kenneth D Mandl, Cecily J Wolfe

In *PLoS Medicine*, volume 3, issue 11, doi:10.1371/journal.pmed.0030502:

Figure 1 from this correspondence was transposed with Figure 1 from the related correspondence by Viboud and colleagues (“Air Travel and the Spread of Influenza: Important Caveats” doi:10.1371/journal.pmed.0030503).

The legend published with Figure 1 (“Influence of United States Airline Volume on Influenza Spread and the Timing of Yearly Transmission over 30 Influenza Seasons”) is correct. However, the figure itself should have appeared as shown below.

**Figure 1:**
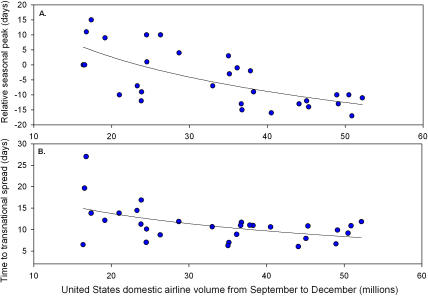
Influence of United States Airline Volume on Influenza Spread and the Timing of Yearly Transmission over 30 Influenza Seasons

